# *In Vivo* 6-([^18^F]Fluoroacetamido)-1-hexanoicanilide PET Imaging of Altered Histone Deacetylase Activity in Chemotherapy-Induced Neurotoxicity

**DOI:** 10.1155/2018/3612027

**Published:** 2018-03-20

**Authors:** Nobuyoshi Fukumitsu, Skye Hsin-Hsien Yeh, Leo Garcia Flores II, Uday Mukhopadhyay, Daniel Young, Kazuma Ogawa, Hwan-Jeong Jeong, William Tong, Juri G. Gelovani

**Affiliations:** ^1^Proton Medical Research Center, University of Tsukuba, Tsukuba, Japan; ^2^Brain Research Center, National Yang-Ming University, Taipei, Taiwan; ^3^Pediatrics Research, UT MD Anderson Cancer Center, Houston, TX, USA; ^4^Cyclotron Research Facility, Center for Advanced Biomedical Imaging, UT MD Anderson Cancer Center, Houston, TX, USA; ^5^Institute for Frontier Science Initiative, Kanazawa University, Kanazawa, Japan; ^6^Department of Nuclear Medicine, Chonbuk National University Medical School & Hospital, Jeonju, Republic of Korea; ^7^Oncology, Neurosurgery, OBGYN, and Biomedical Engineering, Wayne State University, Detroit, MI, USA

## Abstract

**Background:**

Histone deacetylases (HDACs) regulate gene expression by changing histone deacetylation status. Neurotoxicity is one of the major side effects of cisplatin, which reacts with deoxyribonucleic acid (DNA) and has excellent antitumor effects. Suberoylanilide hydroxamic acid (SAHA) is an HDAC inhibitor with neuroprotective effects against cisplatin-induced neurotoxicity.

**Purpose:**

We investigated how cisplatin with and without SAHA pretreatment affects HDAC expression/activity in the brain by using 6-([^18^F]fluoroacetamido)-1-hexanoicanilide ([^18^F]FAHA) as a positron emission tomography (PET) imaging agent for HDAC IIa.

**Materials and Methods:**

[^18^F]FAHA and [^18^F]fluoro-2-deoxy-2-D-glucose ([^18^F]FDG) PET studies were done in 24 mice on 2 consecutive days and again 1 week later. The mice were divided into three groups according to drug administration between the first and second imaging sessions (Group A: cisplatin 2 mg/kg, twice; Group B: cisplatin 4 mg/kg, twice; Group C: cisplatin 4 mg/kg, twice, and SAHA 300 mg/kg pretreatment, 4 times).

**Results:**

The *Ki* value of [^18^F]FAHA was increased and the percentage of injected dose/tissue g (% ID/g) of [^18^F]FDG was decreased in the brains of animals in Groups A and B. The *Ki* value of [^18^F]FAHA and % ID/g of [^18^F]FDG were not significantly different in Group C.

**Conclusions:**

[^18^F]FAHA PET clearly showed increased HDAC activity suggestive of cisplatin neurotoxicity* in vivo*, which was blocked by SAHA pretreatment.

## 1. Introduction

Acetylation and deacetylation on different positions of the N-terminal tail of core histones by histone acetylases (HATs) and histone deacetylases (HDACs) change the nucleosomal conformation of cells [1]. Under normal conditions, the protein concentration and enzymatic activities of HATs and HDACs are carefully balanced. Such equilibrium regulates cellular homeostasis and gene expression to facilitate normal cell function and activity. However, disrupted equilibrium with stronger activity in the deacetylase system leads to transcriptional repression of a diverse set of genes [2, 3].

In recent years, the functional diversity of the HDAC family in the nervous system has been elucidated. A strong association between DNA and histones restricts access by transcription factors and therefore represses gene transcription. Addition of acetyl groups to histones reduces attractive forces between positively charged histone proteins and the negatively charged DNA phosphate backbone, resulting in a more relaxed and accessible chromatin structure. Histone modifications cause dynamic changes in chromatin structure such as transcriptional repression, cell cycle progression, differentiation, and DNA replication that help regulate neuronal gene expression [4–6].

The introduction of cisplatin revolutionized the treatment of certain cancers, and it is now an important component of chemotherapy regimens for lung cancer [7], ovarian cancer [8], head and neck tumors [9], and germ cell tumors [10]. Despite cisplatin's excellent efficacy, it also has several side effects including neurotoxicity, nausea, vomiting, depression, anxiety, visuoperceptual and psychomotor issues, slowed reaction time, decreased verbal conceptualization, short-term memory loss, and weakened attention and executive process [11].

HDAC inhibitors are emerging anticancer agents [12–14] and promising for the treatment of cerebral ischemia, spinal muscular atrophy, and neurodegenerative conditions such as Alzheimer's disease, Parkinson's disease, and Huntington's disease [6, 15]. HDAC inhibitors have been implicated in transcriptional repression, cell cycle progression, differentiation, and DNA replication, as well as the response to DNA damage [16]. Interestingly, some reports have pointed out that the HDAC inhibitors may be neuroprotective against cisplatin-mediated neurotoxicity. Arany et al. reported that trichostatin A (TSA) restored cAMP response element binding protein- (CREB-) mediated transcription after cisplatin activated epidermal growth factor receptor (EGFR)/Ras/extracellular signal-regulated kinase (ERK) signaling in mouse proximal tubule cells [17]. Dong and colleagues proved that TSA and SAHA prevented cisplatin-induced caspase activation by blocking Bax translocation, leading to cytochrome c release from the mitochondria. Their results also indicated that SAHA blocked cisplatin-induced phosphorylation of the DNA damage response kinase Chk2, and SAHA suppressed cisplatin-induced p53 activation but enhanced apoptosis in HCT116 colon cancer cells [18]. Interestingly, Layman reported that systemic SAHA administration offers almost complete protection against hair cell loss from acute ototoxic insult (a side effect of cisplatin) via the increased expression of prosurvival genes Cdkn1a (p21) and Hspa1a (Hsp70) and decreased expression of the proapoptosis gene Bcl2l11 (Bim) [19].

Our group has developed the first PET HDAC IIa radiotracer, 6-([^18^F]fluoroacetamido)-1-hexanoicanilide ([^18^F]FAHA), that can be used to image whole-body HDAC IIa expression and tumor activity [20]. We demonstrated that the rapid [^18^F]FAHA accumulation in the brains of rats and rhesus macaques and the rate of [^18^F]FAHA accumulation were dose-dependently inhibited by the pan-HDAC inhibitor, SAHA (vorinostat). Considering the rapid metabolism of [^18^F]FAHA to [^18^F]FACE in blood, we also developed a multicompartmental pharmacokinetic model with two simultaneous blood input functions to estimate the *Ki* value of [^18^F]FAHA [21, 22]. Significant brain accumulation of [^18^F]FAHA was previously confirmed in a mouse model of NNK (4-[methylnitrosamino]-1-[3-pyridyl]-1-butanone)-induced lung cancer [23] and the baboon brain [24, 25]. These reports indicate the specific interaction of [^18^F]FAHA with HDAC 4 and 5 in the nucleus accumbens, amygdala, hippocampus, and periaqueductal grey matter (all HDAC IIa-rich brain regions) [26]. Indeed, [^18^F]FAHA is the only PET tracer that measures the levels of HDAC IIa expression and activity* in vivo*.

We aimed to investigate (1) whether SAHA attenuates acute cisplatin-induced neurotoxicity in the brain and (2) if our novel imaging biomarker [^18^F]FAHA is suitable to monitor HDAC IIa activity/expression alterations in the brain during treatment with cisplatin or other anticancer drugs.

## 2. Materials and Methods 

### 2.1. Animals

Eight-week-old male athymic nude mice (*n* = 24, Charles River Laboratories) were used in all the studies. The animals were kept at a room temperature of 25°C on a 12 h light/dark cycle and had free access to a standard pellet diet (Lab Diet, Richmond, IN) and tap water. All animal protocols were approved by the Institutional Animal Care and Use Committee of the UT MD Anderson Cancer Center (IACUC Number 03-05-01832).

### 2.2. Drug Administration

The athymic nude mice (8 weeks, Charles River Laboratories) were divided into three groups (8 mice each) according to drug administration: Group A: intraperitoneal (IP) injections of 0.1 mL of cisplatin solution in saline at a concentration of 2 mg/kg cisplatin, twice; Group B: IP injections of 0.1 mL of cisplatin solution in saline at a concentration of 4 mg/kg cisplatin, twice; Group C: IP injections of 0.1 mL of cisplatin solution in saline at a concentration of 4 mg/kg cisplatin, twice, and SAHA 300 mg/kg (10% DMSO in 0.1 mL saline), four times. Two sets of two SAHA injections each followed by cisplatin injections at 12 h intervals were given over 5 days. IP drug administration was performed for 5 days between the first and second [^18^F]FAHA and [^18^F]FDG PET scans (Figure S1).

### 2.3. Radiosynthesis

We performed [^18^F]FAHA radiosynthesis as described in our earlier study [20]. The radiochemical yield was 20% decay corrected, and specific activity > 2 GBq/*μ*mol at the end of the synthesis. The overall radiochemical purity was >99%. [^18^F]FDG was purchased from Cyclotope Inc. (Houston, TX), and the specific activity was estimated as >74 GBq/*μ*mol.

### 2.4. Radiolabeled Metabolites in Plasma

As our preliminary study, we examined the radiolabeled metabolite of [^18^F]FAHA in plasma. The protocols for plasma and serum preparation and analysis were described by Yeh et al. [22]. According to the metabolic analysis procedure, the amount of blood withdrawn for each time point should be at least 200 *μ*L. We therefore used rats instead of mice since it was difficult to withdraw sufficient blood from the mouse tail vein.

Sprague Dawley rats (*n* = 6) were used to estimate the unmetabolized-to-metabolized ratio of [^18^F]FAHA in plasma using the following procedure. Selected blood samples from different time points after [^18^F]FAHA administration were collected and centrifuged. The radioactivity concentrations in whole blood and plasma were counted using a gamma counter (Cobra, Packard, CT). Plasma was extracted and mixed with 3x volumes of acetonitrile and analyzed by high-performance liquid chromatography (HPLC) (Agilent 1100, Santa Clara, CA; Supelcogel C-610H column, Sigma-Aldrich, St. Louis, MO) with a mobile phase of 35% MeCN/phosphoric acid in water at 0.6 ml/min. Fractions of [^18^F]FAHA (parent compound) and [^18^F]FACE (metabolite) were calculated for each sample based on the area under each peak. The total radioactivity in plasma was expressed as percentage of injected dose/mL (% ID/mL) and plotted against time since [^18^F]FAHA injection. The fraction values of [^18^F]FAHA and [^18^F]FACE were calculated based on the results of radio-HPLC analysis and plotted against time. The average radioactivity concentration ratio (plasma-to-blood) and fraction results were applied to the PET quantification, which is described in further detail below.

### 2.5. PET/CT Imaging

The mice (*n* = 24) were anesthetized with isoflurane (2% in oxygen). [^18^F]FAHA (day 1) and [^18^F]FDG (day 2) PET studies were done on two consecutive days and repeated 1 week later on a PET/CT scanner Inveon (Siemens Preclinical Solutions, Knoxville, TN). Food was removed overnight before [^18^F]FDG PET studies. Dynamic [^18^F]FAHA PET studies were acquired for 30 minutes after intravenous (IV) administration of [^18^F]FAHA (7.4 MBq, in 100 *μ*L of saline). Static [^18^F]FDG PET studies for 10 min were acquired 45 min after IV administration of [^18^F]FDG (7.4 MBq, in 100 *μ*L of saline).

[^18^F]FAHA or [^18^F]FDG PET images were reconstructed using a two-dimensional ordered subsets expectation maximization algorithm. PET and CT image fusion and image analyses were performed using Inveon Research Workplace software (Siemens Preclinical Solutions, Knoxville, TN). The CT imaging parameters were X-ray voltage of 80 kVp, anode current of 500 *μ*A, and an exposure time of 300–350 milliseconds for each of the 360 rotational steps. Images were reconstructed using the Shepp Logan algorithm.

### 2.6. Imaging Analysis

A whole-brain region of interest (ROI) was manually drawn on axial PET/CT coregistration images acquired 20–30 min after injection of [^18^F]FAHA or 40 min after injection of [^18^F]FDG. Region placement was by reference to an MRI rodent brain template (PMOD Technologies, Zurich, Switzerland) and a rodent brain atlas.

Dynamic [^18^F]FAHA or static [^18^F]FDG PET images were summed using a rigid transformation algorithm and a normalized mutual method after running a reslicing process (PMOD Technologies). The regional radioactivity concentrations (KBq/mL) of [^18^F]FAHA or [^18^F]FDG PET were estimated from the maximum pixel values within each ROI, expressed as percentage of injected dose/tissue g (% ID/g).

### 2.7. Pharmacokinetic Modeling

#### 2.7.1. Simplified Graphic Analysis: Patlak Plot

The brain and thoracic aorta time-activity data from the dynamic PET scans of [^18^F]FAHA was used for Patlak plot analyses.

(1) $$\begin{array}{c} \frac{R\left(t\right)}{C\left(t\right)}=\frac{Ki\int_{0}^{t}C\left(t\right)dt}{\left(C\right)t}+V_{0}, \end{array}$$

where *t* is time, *R*(*t*) is the mean count of the brain, *C*(*t*) is the mean count of the blood, *Ki* is the clearance determining the rate of entry into the brain, and *V*_0_ is the distribution volume.

The time between injection and the start of the linear phase in the Patlak plot was 4–6 min. Using the data from the start of the linear phase, an accurate linear fit was observed from 6–8 min up to 18–20 min. The slope of the Patlak plot represents the influx rate constant *Ki*.

#### 2.7.2. Pharmacokinetic Modeling

A detailed explanation of the pharmacokinetic model of [^18^F]FAHA-derived radioactivity has been published previously [22]. Briefly, the time-activity curve (TAC) of whole blood was measured from the ROI placed on the carotid artery with the corrected plasma-to-whole blood radioactivity concentration ratio. Then, the fractional values of radioactivity concentrations of [^18^F]FAHA and [^18^F]FACE measured from radio-metabolite analysis (described above) were applied to the image-derived plasma TAC to obtain fraction-corrected TACs for [^18^F]FAHA and [^18^F]FACE, respectively.

Importantly, we performed a separate dynamic PET imaging study with [^18^F]FACE alone to calculate the rate constants *k*1_FACE_, *k*2_FACE_, and *k*3_FACE_ for the rodent. The dynamic imaging-derived [^18^F]FACE input function was corrected for the plasma-to-whole blood ratio and metabolite fraction as described above. The TACs of [^18^F]FACE-derived radioactivity of brain regions were fitted to the irreversible two-tissue compartment model to estimate the rate constants *k*1_FACE_, *k*2_FACE_, and *k*3_FACE_.

Subsequently, the rate constants *k*1_FACE_, *k*2_FACE_, and *k*3_FACE_ obtained from the dynamic PET/CT imaging study with [^18^F]FACE alone were used as fixed rate constants in the model of [^18^F]FAHA-derived radioactivity brain accumulation (Figure S2).

Compartmental modeling, pharmacokinetic analyses, and imaging processing were accomplished using PMOD 3.7 software (PMOD Technologies). [^18^F]FDG uptake of the whole brain was expressed as % ID/g.

### 2.8. Immunohistochemistry (IHC)

Paraffin-embedded sections were cut at 5 *μ*m thickness, deparaffinized, and rehydrated. Antigen retrieval was carried out by microwaving in 10 mM citrate buffer (pH 6.0) at 100°C for 10 min. Sections were washed and then incubated in 3% hydrogen peroxidase for 15 min at room temperature, followed by incubation in blocking solution for 60 min at room temperature.

The primary antibodies employed were acetyl-histone H2A (Lys5), acetyl-histone A2B (Lys20), acetyl-histone A3 (Lys9), and acetyl-histone H4 (Lys8) (all from Cell Signaling Technology, Danvers, MA). All antibodies were employed at a dilution of 1 : 50 and incubated overnight at 4°C. Biotinylated secondary antibodies and 3,3-diaminobenzidine from the Vectastain Elite kit (Vector Laboratories, Burlingame, CA) were used according to the manufacturer's protocol. Microscopic evaluations of immunostained sections were performed using a BX51 microscope equipped with a DP71 digital camera (Olympus, Tokyo, Japan).

A semiquantitative assessment of protein expression was made based on the number of cells showing nuclear expression of each AH3 marker over five nonoverlapping microscopic fields (at ×100 microscope objective magnification), as follows: 0 = absent, less than 5% immunopositive neurons seen; 1 = rare, 10–20% immunopositive neurons per field; 2 = mild, 20–40% mildly or moderately positive neurons per field; 3 = moderate, 40–60% moderately or strongly positive neurons per field; and 4 = strong, more than 80% strongly positive neurons per field. A percentage score for each case was calculated as follows: actual rating × 100/maximal score (i.e., a rating value of 4).

(2) Percentage  of  positive  signal=Sum  of  score of  the  groupNumber  of  case×maximal  score  4×100%.

### 2.9. Animal Behavior Test

Open field and *Y*-maze tests were performed for Groups B and C, 1 day before the first and second PET scans.

### 2.10. Open Field Test

Significant cisplatin neurotoxicity occurs during or after chemotherapy [27], and cisplatin-treated mice exhibit less exploratory behavior after treatment [28]. We tested whether the HDAC inhibitor SAHA could protect against damage induced by cisplatin.

Locomotor and rearing assessments are widely used to test rodent exploratory behavior, and both can be measured in an open field test. The open field was made of plywood and surrounded by 25 cm high walls. The floor of the open field was made of black cardboard (*l* × *w*, 30 × 30 cm) and divided into 9 squares (3 rows of 3). Each mouse was placed individually at the center of the apparatus and observed for 5 min to record locomotor (number of segments crossed with the four paws) and rearing activities (number of times rearing on the hind limbs) [29].

### 2.11. *Y*-Maze Test

Spontaneous alternation behavior in a *Y*-maze task was recorded to evaluate spatial reference memory, according to methods described by Moriguchi et al. [30]. The *Y*-maze apparatus was made of three identical black cardboard pieces (*l* × *w* × *h*, 30 × 8 × 10 cm). Mice were placed at the end of one fixed arm and allowed to move freely through the maze for 5 min. The series of arm entries was recorded visually. Three consecutive choices were defined as an alternation. The alternation behavior (alternation [%]) was calculated as the ratio of actual alternations to possible alternations (defined as the number of arm entries minus two) multiplied by 100.

### 2.12. Statistical Analysis

Values are expressed as mean ± standard deviation. The *Ki* value of [^18^F]FAHA, % ID/g of [^18^F]FDG of the brain, locomotor, rearing activities, and alternation (%) before and after drug (cisplatin with and without SAHA pretreatment) administration were all investigated using paired *t*-test.

## 3. Results

### 3.1. PET Study

#### 3.1.1. [^18^F]FAHA PET

**Figure 1:**
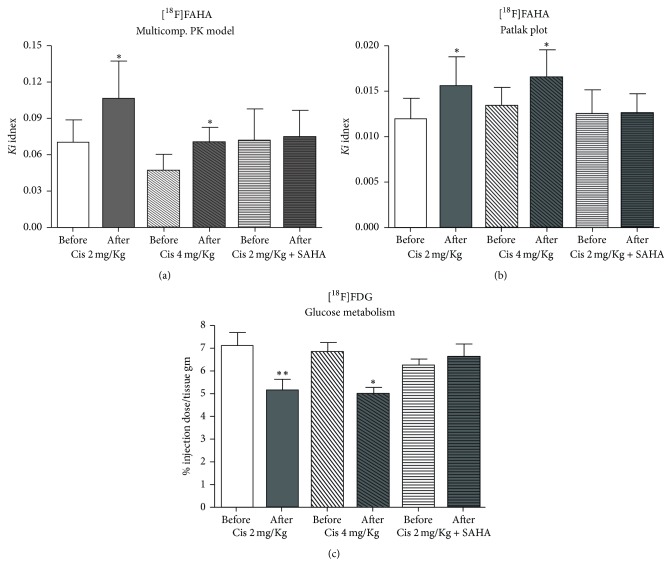
Comparison of *Ki* values of [^18^F]FAHA in the brain calculated from multicompartmental pharmacokinetic model and graphical analyses before and after drug administration. (a) The multicompartmental pharmacokinetic model and (b) Patlak plot both indicated that *Ki* was significantly increased after cisplatin administration in Groups A (Cis 2 mg/kg) and B (Cis 4 mg/kg), but there was no difference in Group C (Cis 4 mg/kg + SAHA 300 mg/kg). (c) In Groups A and B, the radioactivity of accumulated [^18^F]FDG was significantly decreased after cisplatin administration; however, no differences were observed in Group C. ^*∗*^*P* < 0.05; ^*∗∗*^*P* < 0.01.

**Figure 2:**
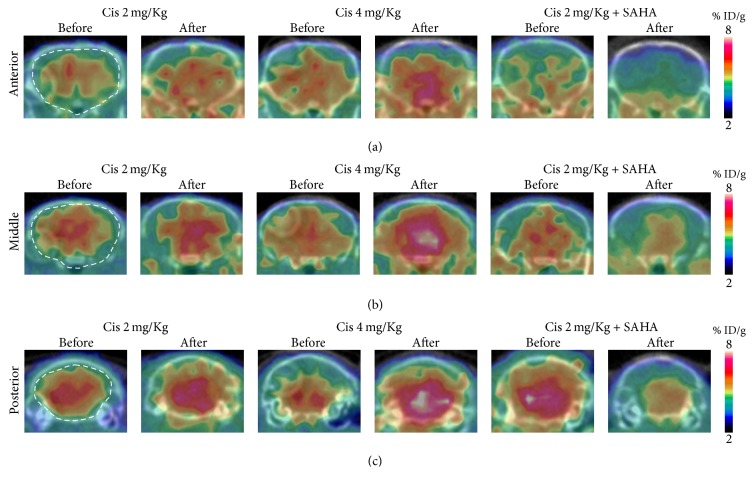
Coronal brain image of [^18^F]FAHA before and after drug administration. [^18^F]FAHA PET/CT images summed over the last 10 min of the study (20–30 min after [^18^F]FAHA injection). (a) Anterior, (b) middle, and (c) posterior part of the brain. Brain [^18^F]FAHA radioactivity was significantly increased after cisplatin administration.

The *Ki* value of [^18^F]FAHA of the brain was significantly increased after cisplatin administration in Groups A and B (Figures 1(a), 1(b), and 2). In Group C, there was a lower *Ki* value of [^18^F]FAHA of the brain after cisplatin administration. The *Ki* value of [^18^F]FAHA of the brain was not significantly altered after cisplatin with SAHA administration. Graphical analyses (Patlak plot) and the multicompartmental pharmacokinetic model showed similar quantitative results.

#### 3.1.2. [^18^F]FDG PET

**Figure 3:**
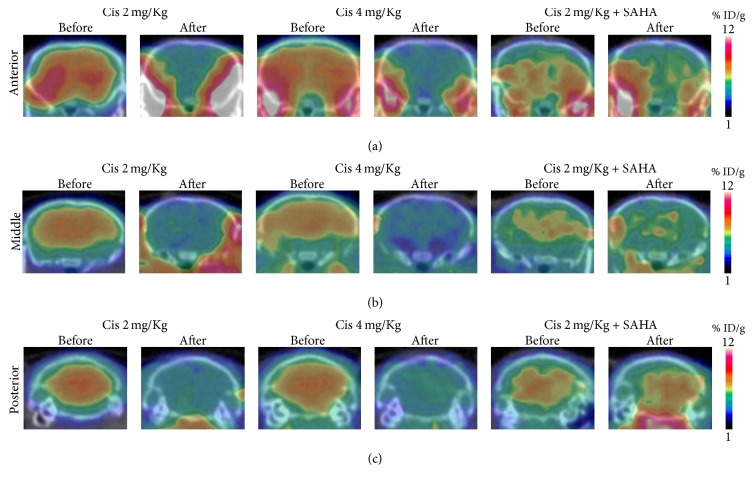
Coronal brain image of [^18^F]FDG before and after drug administration. The [^18^F]FDG image represents 45–55 min after injection. Image presentation is the same as in Figure 2. Brain [^18^F]FAHA radioactivity was decreased after cisplatin administration.

The accumulated brain radioactivity of [^18^F]FDG was significantly decreased after cisplatin administration in Groups A and B (Figures 1(c) and 3). In Group C, the accumulated brain radioactivity of [^18^F]FDG was not significantly changed after cisplatin with SAHA administration (Figures 1(c) and 3).

### 3.2. IHC

**Figure 4:**
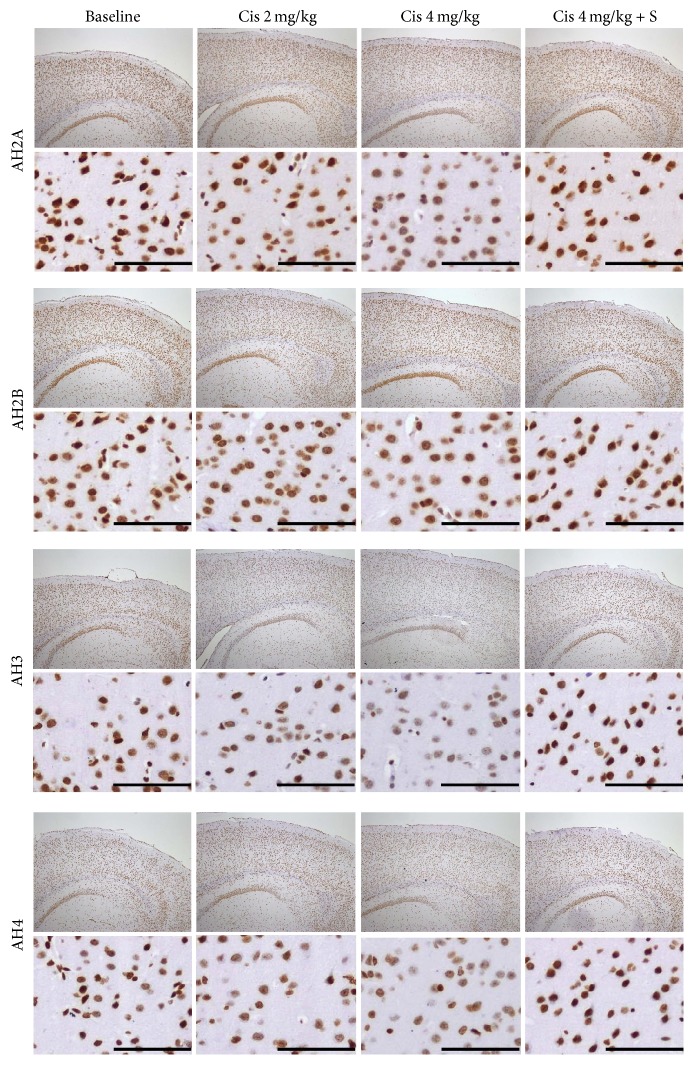
IHC for AH2A, AH2B, AH3, and AH4 in the cerebral cortex. Original magnifications, upper: ×40, lower: ×200 in the parietal cortex. AH3 staining was suppressed in a dose-dependent manner in the cerebral cortex of mice in Groups A (Cis 2 mg/kg) and B (Cis 4 mg/kg) and was almost equal in Group C (Cis 4 mg/kg + SAHA 300 mg/kg) compared with a control mouse. Scale bar, 100 *μ*m.

**Figure 5:**
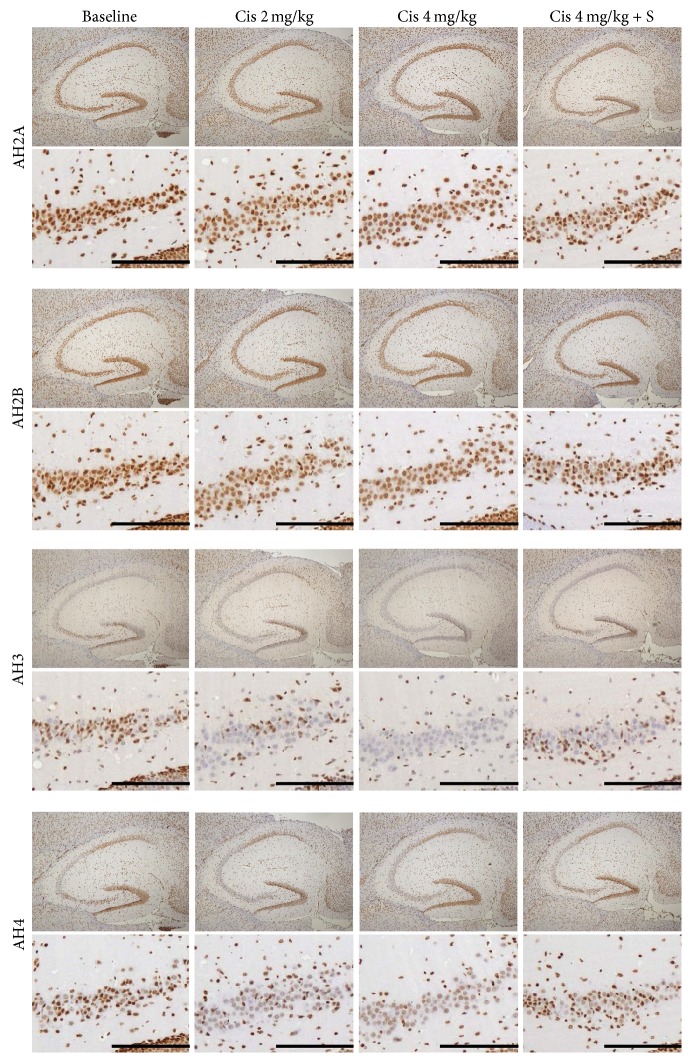
IHC for AH2A, AH2B, AH3, and AH4 in the hippocampus. Image presentation is the same as in Figure 4. Original magnification, upper: ×40 in the hippocampus, lower: ×100 in the Ca3. AH3 staining was suppressed in a dose-dependent manner in the CA3 and dentate gyrus of mice in Groups A (Cis 2 mg/kg) and B (Cis 4 mg/kg) and was almost equal in Group C (Cis 4 mg/kg+ SAHA 300 mg/kg), compared with a control mouse. Scale bar, 100 *μ*m.

**Figure 6:**
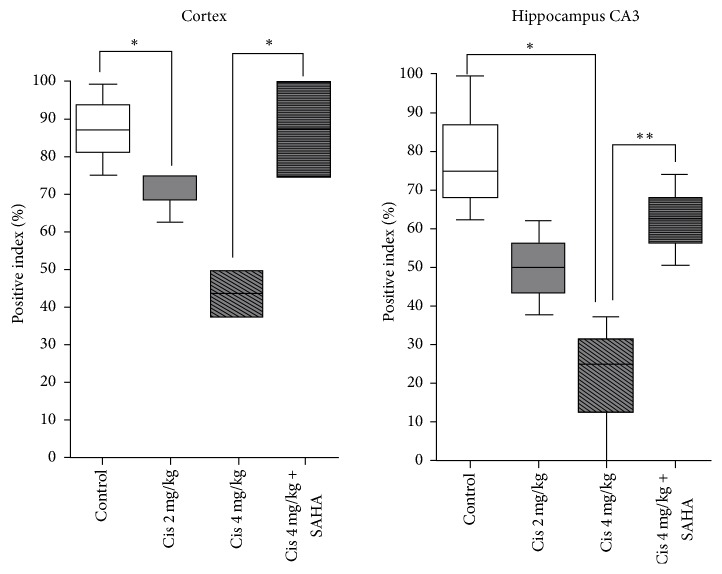
Corresponding values stained with AH3 in the cortex (Figure 4) and hippocampus CA3 (Figure 5). ^*∗*^*P* < 0.05; ^*∗∗*^*P* < 0.01.

In the cortex, AH2A, AH3, and AH4 staining were decreased in a dose-dependent manner with cisplatin in Groups A and B and were almost equal in Group C compared to control. The change in AH3 staining was dramatic in the cerebral cortex. In the hippocampus, AH3 and AH4 staining were decreased with cisplatin in a dose-dependent manner in Groups A and B, while they were almost equal in Group C in the cornu ammonis 3 (CA3) and dentate gyrus compared to control. The changes of AH2A and AH2B were minimal. Representative IHC images are shown of AH3 staining in the cerebral cortex (Figure 4) and hippocampus (Figure 5). AH3 immunoreactivity in the cortex and hippocampal CA3 is shown in Figure 6. It was significantly lower in the CA3 of Groups A and B compared to the control, whereas Group C had moderate immunoreactivity in cortex. Hippocampal immunoreactivity of AH3 showed a similar pattern.

### 3.3. Animal Behavior

**Figure 7:**
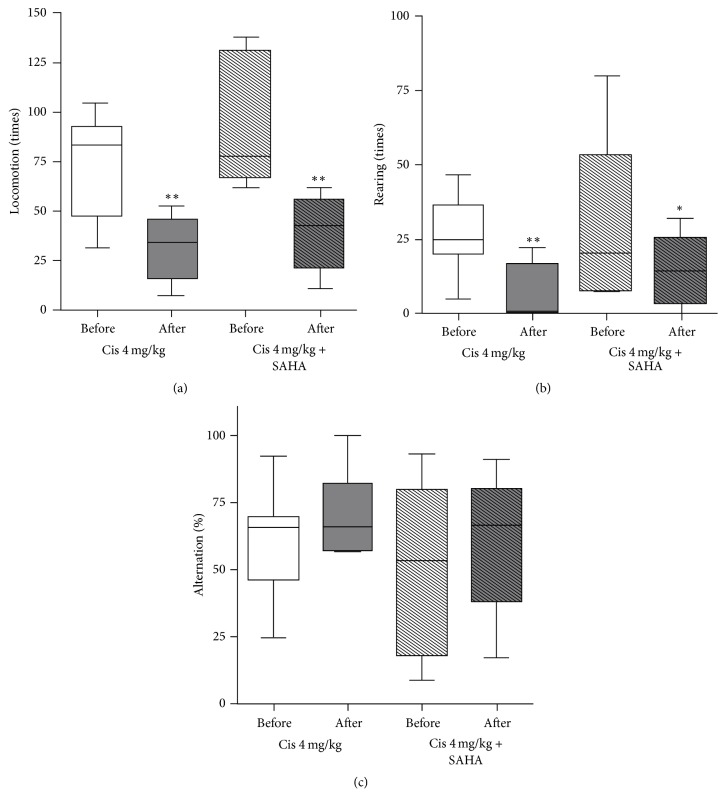
Animal behavior test: (a) locomotion, (b) rearing activity, and (c) alteration. Locomotion and rearing activity decreased after cisplatin administration with and without SAHA pretreatment. Alternation (%) was not significantly changed after cisplatin administration with and without SAHA pretreatment. ^*∗*^*P* < 0.05; ^*∗∗*^*P* < 0.01.

In Group B (cisplatin 4 mg/kg), locomotor and rearing activity were significantly decreased after cisplatin administration (72.8 ± 27.0 before cisplatin administration versus 31.1 ± 16.5 after cisplatin administration, *p* = 0.003; 26.8 ± 13.5 versus 8.4 ± 9.1, *p* = 0.009, resp., Figures 7(a) and 7(b)). Alternation (%) was not significantly changed (60.1 ± 20.9 versus 70.9 ± 17.0, *p* = 0.300, Figure 7(c)).

In Group C (cisplatin 4 mg/kg with SAHA 300 mg/kg), locomotor and rearing activity were significantly decreased after cisplatin with SAHA administration (94.3 ± 33.6 before cisplatin with SAHA administration versus 38.5 ± 19.5 after cisplatin with SAHA administration, *p* = 0.008; 31.0 ± 28.1 versus 14.8 ± 12.3, *p* = 0.048, resp., Figures 7(a) and 7(b)). The alternation (%) was not significantly changed (60.2 ± 31.3 versus 59.4 ± 26.5, *p* = 0.948, Figure 7(c)).

## 4. Discussion

The mechanism of cisplatin-induced neurotoxicity is complicated and has not been fully elucidated. However, the major neurotoxic mechanism is thought to involve p53, Bcl-2, caspases 3 and 7, the mitogen-activated protein kinase family, and the FasR pathway [31–34]. Cisplatin reacts with DNA, and its cytotoxic effects are due to the generation of 1,2-intrastrand cross-links between adjacent purines in d(GpG) and d(ApG) sequences [35, 36]. Although histone proteins play an important role in DNA transcription, the participation of HATs and HDACs that form part of the transcription initiation complex with transcription factors and regulate histone proteins has not been fully investigated in the brain. We found that cisplatin treatment was followed by an increased *Ki* value of [^18^F]FAHA in the brain. In contrast, the % ID/g of [^18^F]FDG was decreased in the brain, and animal behavior was suppressed following cisplatin administration. This suggests that cisplatin causes excessive HDAC deacetylation in the brain, subsequently decreasing the transcription of neuronal DNA. In addition, the IHC results support our hypothesis of an imbalance between HAT acetylation and HDAC deacetylation in the brain of cisplatin-treated mice.

How HDAC inhibitors work with cisplatin in normal cells has not been elucidated, despite the fact that HDAC inhibitors could potentially be used as anticancer drugs. Some reports have stated that HDAC inhibitor pretreatment inhibits cisplatin-mediated toxicity and promotes renal cell survival [17, 18]. However, to the best of our knowledge, there has been no report on how HDAC inhibitors work with cisplatin in the brain. We tested the hypothesis that HDAC inhibitor pretreatment may block the DNA damage response in the normal brain and assessed whether it can be detected using* in vivo *PET scanning. No significant change was observed in the mean *Ki* value of [^18^F]FAHA, and six out of eight mice had lower values compared to the study before cisplatin with SAHA pretreatment. For [^18^F]FDG PET, the % ID/g of brain [^18^F]FDG showed almost no change. The trend of the change of the *Ki* value of [^18^F]FAHA and % ID/g of [^18^F]FDG in the brains of Group C mice was obviously different from those in Groups A and B. Moreover, the result of Groups B and C clearly demonstrated that SAHA pretreatment inhibited the increase in [^18^F]FAHA uptake and the decrease in [^18^F]FDG uptake in the brains of cisplatin-treated mice. This suggests that SAHA blocks excess HDAC deacetylation and neuronal activity suppression in the brain following cisplatin administration. The IHC results also support our hypothesis that SAHA pretreatment blocks unbalanced excess HDAC deacetylation in the brains of cisplatin-treated mice. Taken together, our findings indicate that SAHA pretreatment prevents cisplatin-mediated changes in HAT acetylation and HDAC deacetylation in the brain.

The fundamental unit of chromatin is the nucleosome, composed of DNA wrapped around an octamer of four core histone proteins (H2A, H2B, H3, and H4), each of which possesses a large number and type of modifiable residues [3, 37]. However, DNA contains both survival-associated and death-inducing genes. We do not have enough evidence to identify which histones in survival-associated genes or death-inducing genes reflect [^18^F]FAHA via changes in their acetylation/deacetylation balance. However, our PET results indicate that [^18^F]FAHA activity seems to be strongly reflected by histones in survival-associated genes in the brain. AH3 and AH4 staining are observed during the onset of neuronal apoptosis in neurodegeneration [38]. This is consistent with our finding that AH3 and AH4 show demonstrable staining in a wide area of the brain in cisplatin-administered mice. We consider that [^18^F]FAHA has the strong potential to reflect H3 and H4 deacetylation in survival-associated genes in the brain.

Although SAHA pretreatment blocked histone deacetylation in the brain and contributed to reducing attractive forces between histone proteins and the DNA phosphate backbone, resulting in a more relaxed and accessible chromatin structure, there was no obvious improvement in locomotion. This may be due to the fact that HDAC inhibitors only affect the expression of 2% of mammalian genes [2, 39].

## 5. Conclusion

We performed [^18^F]FAHA and [^18^F]FDG PET to investigate changes in HDAC IIa activity in the brains of mice treated with cisplatin with and without SAHA pretreatment. [^18^F]FAHA PET clearly showed higher HDAC IIa activity after cisplatin administration, and this was blocked by SAHA pretreatment. In contrast, [^18^F]FDG uptake was decreased after cisplatin administration and was blocked by SAHA pretreatment. Therefore, [^18^F]FAHA PET could be a useful* in vivo *pharmacodynamic biomarker of cisplatin neurotoxicity.

## Supplementary Materials

Supplementary 1 Supplementary Figure S1: schematic and graphical representation of the study design. Animals were divided to 3 groups and received intraperitoneal doses of cisplatin 2 mg/kg (Group A) or 4 mg/kg (Group B) or 4 mg/kg with SAHA 300 mg/kg (Group C) (details in the Materials and Methods). PET/CT imaging was performed before and after drug administration. Additionally, Groups B and C were tested with behavioral assays to assess cognitive function one day before the first and second PET/CT studies. After PET/CT imaging, the results were validated by IHC of brain tissue sections.

Supplementary 2 Supplementary Figure S2: illustration of a multicompartmental pharmacokinetic model with two simultaneous blood input functions for [^18^F]FAHA and [^18^F]FACE that was used to estimate the *Ki* values of [^18^F]FAHA in this study.

## References

[1] Minucci S., Pelicci P. G. (2006). Histone deacetylase inhibitors and the promise of epigenetic (and more) treatments for cancer. *Nature Reviews Cancer*.

[2] Saha R. N., Pahan K. (2006). HATs and HDACs in neurodegeneration: a tale of disconcerted acetylation homeostasis. *Cell Death & Differentiation*.

[3] Marks P. (2001). Histone deacetylases and cancer: causes and therapies. *Nature Reviews Cancer*.

[4] Levenson J. M., O'Riordan K. J., Brown K. D., Trinh M. A., Molfese D. L., Sweatt J. D. (2004). Regulation of histone acetylation during memory formation in the hippocampus. *The Journal of Biological Chemistry*.

[5] Bredy T. W. (2007). Histone modifications around individual BDNF gene promoters in prefrontal cortex are associated with extinction of conditioned fear. *Learning Memory*.

[6] Morrison B. E., Majdzadeh N., D’Mello S. R. (2007). Histone deacetylases: Focus on the nervous system. *Cellular and Molecular Life Sciences*.

[7] Spiro S. G. (2004). Chemotherapy versus supportive care in advanced non-small cell lung cancer: improved survival without detriment to quality of life. *Thorax*.

[8] Armstrong D. K. (2006). Intraperitoneal cisplatin and paclitaxel in ovarian cancer. *The New England Journal of Medicine*.

[9] Adelstein D. J. (2004). An intergroup phase III comparison of standard radiation therapy and two schedules of concurrent chemoradiotherapy in patients with unresectable squamous cell head andneck cancer. *Journal of Clinical Oncology*.

[10] Schmoll H. J. (2004). European consensus on diagnosis and treatment of germ cell cancer: a report of the European Germ Cell Cancer Consensus Group (EGCCCG). *Annals of Oncology*.

[11] Troy L. (2000). Cisplatin-based therapy: a neurological and neuropsychological review. *Psychooncology*.

[12] Bolden J. E., Peart M. J., Johnstone R. W. (2006). Anticancer activities of histone deacetylase inhibitors. *Nature Reviews Drug Discovery*.

[13] Marks P. A., Breslow R. (2007). Dimethyl sulfoxide to vorinostat: development of this histone deacetylase inhibitor as an anticancer drug. *Nature Biotechnology*.

[14] Frew A. J., Johnstone R. W., Bolden J. E. (2009). Enhancing the apoptotic and therapeutic effects of HDAC inhibitors. *Cancer Letters*.

[15] Dietz K. C., Casaccia P. (2010). HDAC inhibitors and neurodegeneration: At the edge between protection and damage. *Pharmacological Research*.

[16] Roos W. P., Krumm A. (2016). The multifaceted influence of histone deacetylases on DNA damage signalling and DNA repair. *Nucleic Acids Research*.

[17] Arany I., Herbert J., Herbert Z., Safirstein R. L. (2008). Restoration of CREB function ameliorates cisplatin cytotoxicity in renal tubular cells. *American Journal of Physiology-Renal Physiology*.

[18] Dong G., Luo J., Kumar V., Dong Z. (2010). Inhibitors of histone deacetylases suppress cisplatin-induced p53 activation and apoptosis in renal tubular cells. *American Journal of Physiology-Renal Physiology*.

[19] Layman W. S. (2015). Histone deacetylase inhibition protects hearing against acute ototoxicity by activating the Nf-kappaB pathway. *Cell Death Discovery*.

[20] Mukhopadhyay U., Tong W. P., Gelovani J. G., Alauddin M. M. (2006). Radiosynthesis of 6-([18F]fluoroacetamido)-1-hexanoic-anilide ([18F]FAHA) for PET imaging of histone deacetylase (HDAC). *Journal of Labelled Compounds and Radiopharmaceuticals*.

[21] Sankaranarayanapillai M. (2006). Detection of histone deacetylase inhibition by noninvasive magnetic resonance spectroscopy. *Molecular Cancer Therapeutics*.

[22] Yeh H.-H., Tian M., Hinz R. (2013). Imaging epigenetic regulation by histone deacetylases in the brain using PET/MRI with ^18^F-FAHA. *NeuroImage*.

[23] Tang W. (2014). Targeting histone deacetylase in lung cancer for early diagnosis: (18)F-FAHA PET/CT imagining of NNK-treated A/J mice model. *Am J Nucl Med Mol Imaging*.

[24] Seo Y. J., Muench L., Reid A. (2013). Radionuclide labeling and evaluation of candidate radioligands for PET imaging of histone deacetylase in the brain. *Bioorganic & Medicinal Chemistry Letters*.

[25] Reid A. E., Hooker J., Shumay E. (2009). Evaluation of 6-([(18)F]fluoroacetamido)-1-hexanoicanilide for PET imaging of histone deacetylase in the baboon brain. *Nuclear Medicine and Biology*.

[26] Broide R. S., Redwine J. M., Aftahi N., Young W., Bloom F. E., Winrow C. J. (2007). Distribution of histone deacetylases 1-11 in the rat brain. *Journal of Molecular Neuroscience*.

[27] Amptoulach S., Tsavaris N. (2011). Neurotoxicity caused by the treatment with platinum analogues. *Chemotherapy Research and Practice*.

[28] Ta L. E., Low P. A., Windebank A. J. (2009). Mice with Cisplatin and Oxaliplatin-Induced Painful Neuropathy Develop Distinct Early Responses to Thermal Stimuli. *Molecular Pain*.

[29] Walsh R. N., Cummins R. A. (1976). The open-field test: a critical review. *Psychological Bulletin*.

[30] Moriguchi S., Han F., Nakagawasai O., Tadano T., Fukunaga K. (2006). Decreased calcium/calmodulin-dependent protein kinase II and protein kinase C activities mediate impairment of hippocampal long-term potentiation in the olfactory bulbectomized mice. *Journal of Neurochemistry*.

[31] Donzelli E., Carfì M., Miloso M. (2004). Neurotoxicity of platinum compounds: comparison of the effects of cisplatin and oxaliplatin on the human neuroblastoma cell line SH-SY5Y. *Journal of Neuro-Oncology*.

[32] Rzeski W., Pruskil S., Macke A. (2004). Anticancer agents are potent neurotoxins in vitro and in vivo. *Annals of Neurology*.

[33] Szatmari E., Kalita K. B., Kharebava G., Hetman M. (2007). Role of Kinase Suppressor of Ras-1 in Neuronal Survival Signaling by Extracellular Signal-Regulated Kinase 1/2. *The Journal of Neuroscience*.

[34] Gozdz A. (2008). Cisplatin-mediated activation of extracellular signal-regulated kinases 1/2(ERK1/2) by inhibition of ERK1/2 phosphatases. *J Neurochem*.

[35] Nafisi S., Norouzi Z. (2009). A comparative study on the interaction of cis- and trans-platin with DNA and RNA. *DNA Cell Biol*.

[36] Jamieson E. R., Lippard S. J. (1999). Structure, Recognition, and Processing of Cisplatin-DNA Adducts. *Chemical Reviews*.

[37] MacDonald J. L., Roskams A. J. (2009). Epigenetic regulation of nervous system development by DNA methylation and histone deacetylation. *Progress in Neurobiology*.

[38] Rouaux C., Jokic N., Mbebi C., Boutillier S., Loeffler J.-P., Boutillier A.-L. (2003). Critical loss of CBP/p300 histone acetylase activity by caspase-6 during neurodegeneration. *EMBO Journal*.

[39] Van Lint C., Emiliani S., Verdin E. (1996). The expression of a small fraction of cellular genes is changed in response to histone hyperacetylation. *Gene Expression*.

