# Supplementary material for: In Vivo 6-([18F]Fluoroacetamido)-1-hexanoicanilide PET Imaging of Altered Histone Deacetylase Activity in Chemotherapy-Induced Neurotoxicity
Source: Contrast Media Mol Imaging. 2018 Mar 20;2018:3612027. doi: 10.1155/2018/3612027 (PMC5884410; doi:10.1155/2018/3612027)
Supplement: Supplementary 1 — Supplementary Figure S1: schematic and graphical representation of the study design. Animals were divided to 3 groups and received intraperitoneal doses of cisplatin 2 mg/kg (Group A) or 4 mg/kg (Group B) or 4 mg/kg with SAHA 300 mg/kg (Group C) (details in the Materials and Methods). PET/CT imaging was performed before and after drug administration. Additionally, Groups B and C were tested with behavioral assays to assess cognitive function one day before the first and second PET/CT studies. After PET/CT imaging, the results were validated by IHC of brain tissue sections. [file 3612027.f1.pdf]

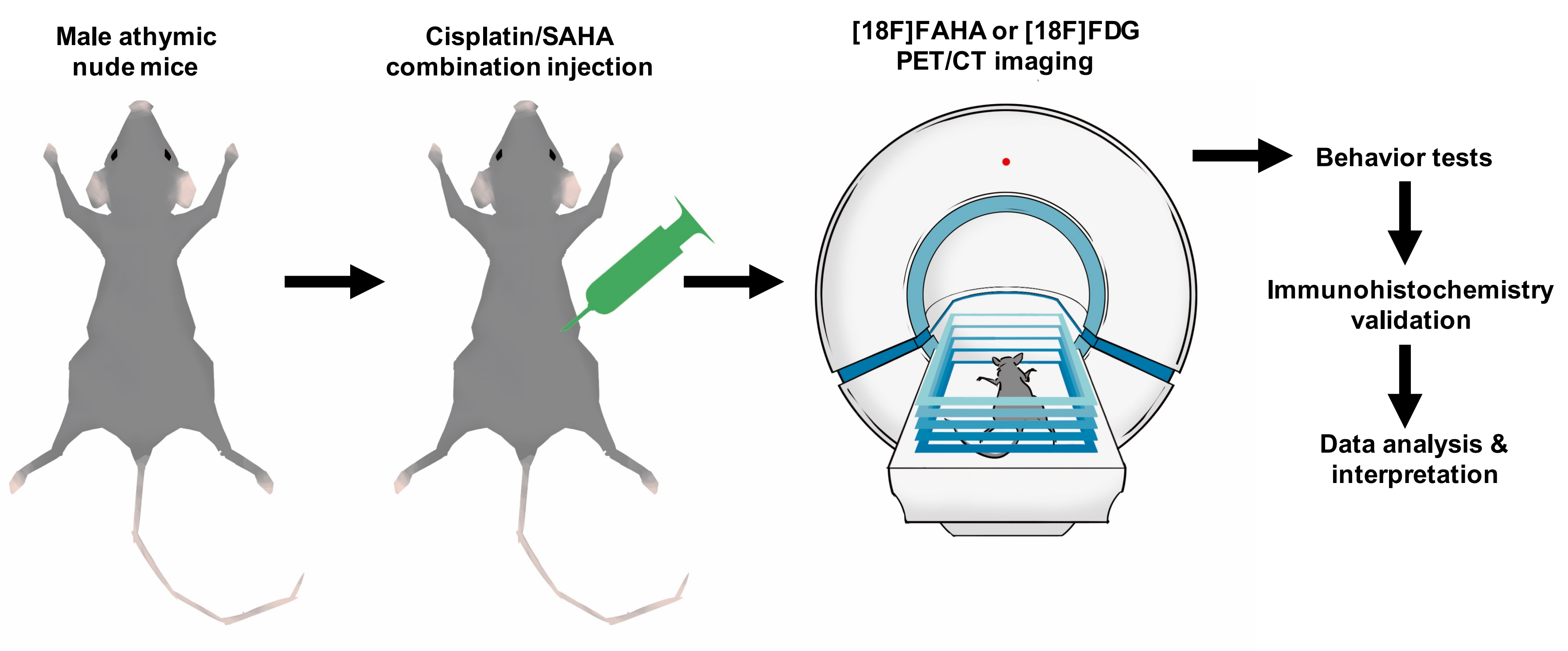
Male athymic nude mice
Cisplatin/SAHA combination injection
[18F]FAHA or [18F]FDG PET/CT imaging
Behavior tests
Immunohistochemistry validation
Data analysis & interpretation
