# Supplementary figures and images for: In Vivo 6-([18F]Fluoroacetamido)-1-hexanoicanilide PET Imaging of Altered Histone Deacetylase Activity in Chemotherapy-Induced Neurotoxicity
Source: Contrast Media Mol Imaging. 2018 Mar 20;2018:3612027. doi: 10.1155/2018/3612027 (PMC5884410; doi:10.1155/2018/3612027)

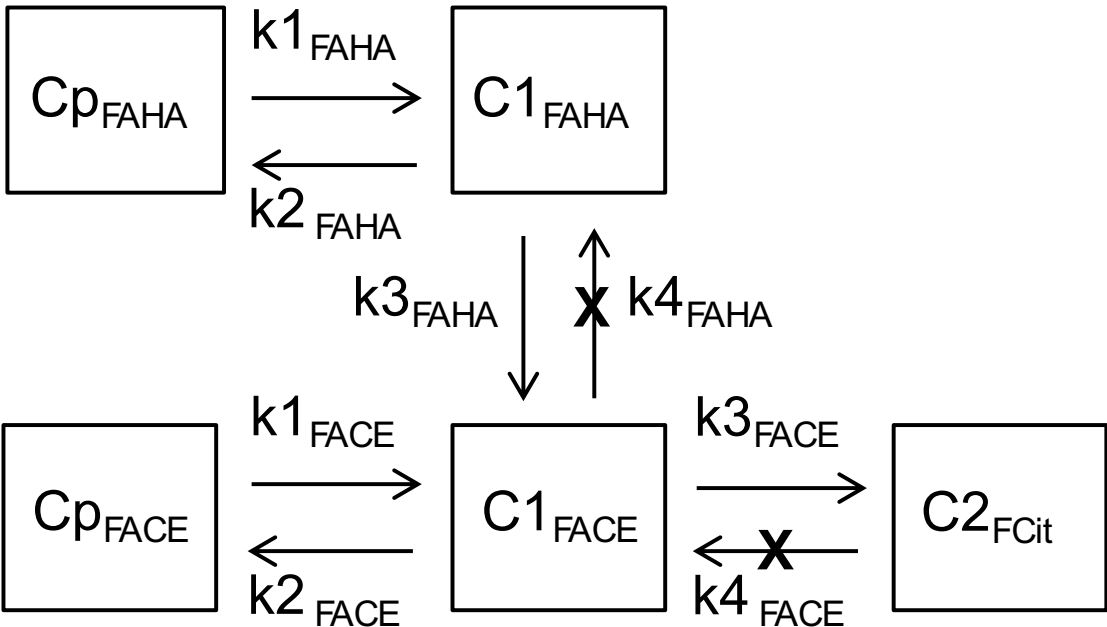

Supplement: Supplementary 2 — Supplementary Figure S2: illustration of a multicompartmental pharmacokinetic model with two simultaneous blood input functions for [18F]FAHA and [18F]FACE that was used to estimate the Ki values of [18F]FAHA in this study. [file 3612027.f2.pdf]
